# Efficient differential privacy enabled federated learning model for detecting COVID-19 disease using chest X-ray images

**DOI:** 10.3389/fmed.2024.1409314

**Published:** 2024-06-03

**Authors:** Rawia Ahmed, Praveen Kumar Reddy Maddikunta, Thippa Reddy Gadekallu, Naif Khalaf Alshammari, Fatma Ali Hendaoui

**Affiliations:** ^1^Computer Science Department, Applied College, University of Ha’il, Ha’il, Saudi Arabia; ^2^School of Computer Science Engineering and Information Systems, Vellore Institute of Technology, Vellore, Tamil Nadu, India; ^3^The College of Mathematics and Computer Science, Zhejiang A&F University, Hangzhou, China; ^4^Division of Research and Development, Lovely Professional University, Phagwara, India; ^5^Center of Research Impact and Outcome, Chitkara University, Rajpura, India; ^6^Mechanical Engineering Department, Engineering College, University of Ha’il, Ha’il, Saudi Arabia; ^7^Computer Science Department, Applied College, University of Ha’il, Ha’il, Saudi Arabia

**Keywords:** COVID-19 detection, decentralized training, adaptive differential privacy, federated learning, convolutional neural network, healthcare data privacy

## Abstract

The rapid spread of COVID-19 pandemic across the world has not only disturbed the global economy but also raised the demand for accurate disease detection models. Although many studies have proposed effective solutions for the early detection and prediction of COVID-19 with Machine Learning (ML) and Deep learning (DL) based techniques, but these models remain vulnerable to data privacy and security breaches. To overcome the challenges of existing systems, we introduced Adaptive Differential Privacy-based Federated Learning (DPFL) model for predicting COVID-19 disease from chest X-ray images which introduces an innovative adaptive mechanism that dynamically adjusts privacy levels based on real-time data sensitivity analysis, improving the practical applicability of Federated Learning (FL) in diverse healthcare environments. We compared and analyzed the performance of this distributed learning model with a traditional centralized model. Moreover, we enhance the model by integrating a FL approach with an early stopping mechanism to achieve efficient COVID-19 prediction with minimal communication overhead. To ensure privacy without compromising model utility and accuracy, we evaluated the proposed model under various noise scales. Finally, we discussed strategies for increasing the model’s accuracy while maintaining robustness as well as privacy.

## 1 Introduction

The global healthcare system faces an unprecedented challenge due to SARS-CoV-2. The COVID-19 pandemic has emerged as a significant global health crisis, impacting millions worldwide and causing widespread economic and societal disruption on a global scale. The rapid spread of the virus has led to the harnessing of cutting-edge technologies for patient data collection, disease prediction, surveillance, and management. COVID-19 disease-related data being generated or collected by the various Internet of Things (IoT) applications are being managed and processed using efficient big data analytics and computational methods such as ML or DL algorithms (1). Diverse healthcare datasets are collected, encompassing epidemiological data (e.g., confirmed cases, deaths, recoveries), clinical records (e.g., symptoms, comorbidities), demographic information (e.g., gender, age), and socio-economic factors (e.g., population density, mobility patterns). However, this data inherently contains sensitive information related to specific patients, regions, or locations (2). Therefore, robust measures are crucial to safeguard data privacy and confidentiality during various activities such as sharing, exchanging, managing, and processing, which often involve multiple entities and tools. Healthcare data privacy standards guarantee that only authorized individuals or organizations have access to a patient’s personal medical information. This protects sensitive information like a patient name, patient address, date of birth, and important medical status being shared without their consent (3). However, traditional centralized systems have major drawbacks, including significant processing time, increased network traffic, and a heightened risk of unauthorized data access.

**Figure 1:**
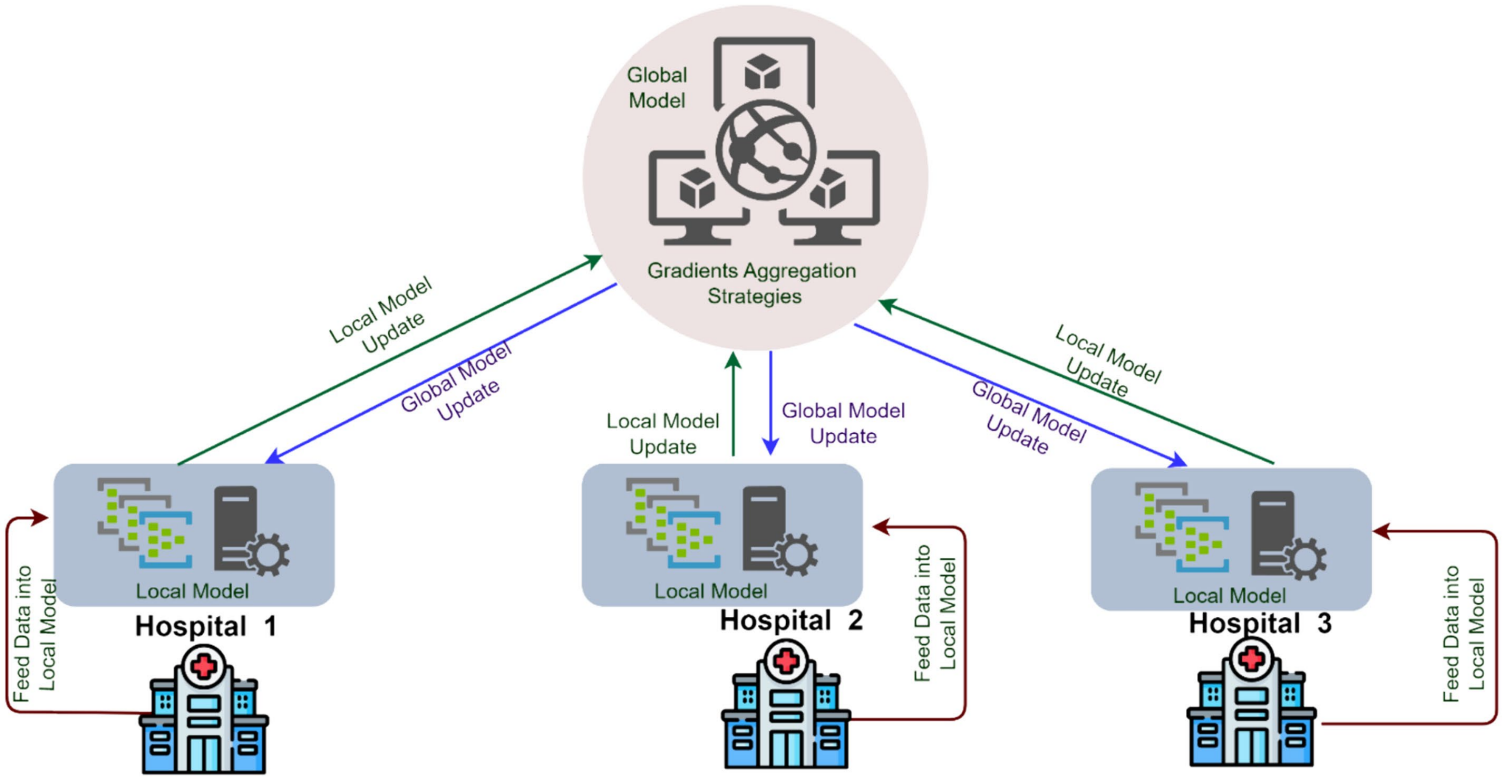
Federated learning in healthcare systems.

Over the years, various methods have been developed for addressing the limitations of centralized architectures. While preserving data privacy and confidentiality through authorized access control. However, recent advances in applied AI technologies provide promising results with distributed learning techniques, resulting in increased data processing. FL is a distributed learning approach in which only model parameters are exchanged between the server and clients over several iterations, rather than actual data being transferred to the server. The clients perform training on their data using the model parameters provided by the server. Throughout this process, initial privacy is provided, and communication costs are reduced. Since the amount of data on clients is less compared to the central data pool, local learning is attained with minimal hardware requirements (4). Figure 1 illustrates the processing of medical data from various hospitals using FL architecture. Although FL achieves privacy through the physical isolation of data, it does not guarantee privacy for local data. During the model transmission process, the server can invert the client’s local information using model gradients, leading to a potential inference attack. Even though FL fulfills the design principles necessary for achieving privacy, but still, the attacker can still steal the private information of a user through the intermediate results of the FL process (5). However, this be addressed in two ways. First, we can consider encryption methods to protect the information flow of intermediate results such as Homomorphic Encryption (HE) (6) and Secure Multi-party Computation (MPC) (7). Secondly, we can consider the perturbation of the original private information, through techniques such as Differential Privacy (DP), which can prevent the revelation of intermediate results (8).

By introducing noise to the original dataset or learning parameters, the DP technique guarantees a high level of privacy protection in data analysis, thus making it impossible for attackers to access sensitive data. Although DP was proposed in 2006, its recent AI applications to improve data security, stabilize the learning process, develop unbiased models, and apply composition in specific AI domains have attracted significant interest from researchers and tech titans such as Google, Microsoft, and Apple (9). These organizations are interested in retrieving statistics from client devices, either by developing applications with Central Differential Privacy (CDP) or Local Differential Privacy (LDP) techniques (10). CDP techniques involve the inclusion of random noise to the actual data after it has been acquired from all clients by a data curator in a central server. However, the LDP mechanism introduces noise before transmitting the data or learning parameter to the central server, guaranteeing privacy from the beginning of data transmission process. Besides applications in ML and DL, DP has also improved the convergence rate by guaranteeing privacy in distributed learning environments (11). An adaptive Differential Privacy Federated Learning Medical IoT (DPFL-MIoT) uses several techniques such as DP, FL, and deep neural networks with adaptive gradient descent to mask model parameters by infusing noise (12).

The main contributions of the work are as follows:

1. We have developed a distributed learning model to predict COVID-19 disease by considering the three different classes of Chest X-Ray images such as COVID, Normal, and Pneumonia.
2. We designed Adaptive Differential Privacy-Enhanced Federated Learning (DPFL) framework with an early-stopping technique to preserve patient data while maintaining utility.
3. We have conducted several experiments to analyze and evaluate the Utility and Privacy of the data, and the impact of the early stopping mechanism on the performance of the proposed DPFL model.

The rest of the paper is organized as follows: Section 2 discusses existing works on FL and AFL using DP. Section 3 presents the proposed FL models with a DP mechanism. A detailed discussion of the experimental setup, dataset, and obtained results are provided in Section 4. Finally, the conclusion and future research directions are discussed in Section 5.

## 2 Literature review

FL revolutionizes ML by decentralizing model training across devices, safeguarding local data privacy. This collaborative model involves a central server managing global parameters and clients with local datasets. Model updates from clients enhance the global model iteratively. FL offers advantages like privacy preservation, reduced communication overhead, and collaborative learning. Challenges include handling heterogeneous data and addressing communication and security concerns. This sets the stage for exploring privacy-preserving mechanisms like Differential Privacy within the FL framework. To reduce the prediction bias and to eradicate the overfitting problems caused by to small dataset, Chen et al. (13) have proposed a DP-based adaptive worker selection algorithm. The proposed framework generated a vulnerability prediction map considering COVID-19 data through various apps using distributed FL models to ensure privacy. Wu et al. (14) suggested an FL model with an adaptive gradient descendent and differential privacy mechanism for a multiparty collaborative environment by ensuring efficient model training with minimal communication cost. Even though, the proposed technique enhances the accuracy and stability of the model but still lacks model convergence efficiency due to hyperparameter fluctuations. Ulhaq et al. (15) have developed a Differential privacy-enabled FL framework for COVID-19 disease diagnosis by ensuring data privacy. The authors have designed and developed the theoretical model, hence the model needs to be implemented for further analysis.

Similarly, Wang et al. (16) have designed a privacy-enhanced disease diagnosis using FL. The proposed model incorporates Variational Autoencoder (VAE), differential privacy noise, and incentive mechanism during the disease diagnosis process in a distributed environment. Simulation results have shown that the accuracy of the global model decreases with an increase in the privacy budget. The privacy requirements of the individuals are not the same, hence the authors Liu et al. (17) have introduced a hybrid differential privacy technique to the existing privacy-friendly FL framework by dividing the user into groups as per their privacy requirements. The adaptive gradient clipping mechanism and improved composition methods of the model will improve the model accuracy by reducing the noise issues. To reduce the impact of noise on the accuracy of the model the authors Yang et al. (18) have proposed Kalman Filter-based Differential Privacy Federated Learning Method (KDP-FL). The Proposed algorithm was tested in a simulated environment; however, the Kalman filter noise reduction method results in better accuracy but increases the computational overhead.

To reduce and nullify the leakage of sematic information of the training data by the Generative Adversarial Networks (GAN), the author’s Zhang et al. (19) have developed a “Federated Differentially Private Generative Adversarial Network (FedDPGAN)” model for the detection of COVID-19 pneumonia, which is aimed to improve the data privacy of the patients. DP-GAN of the proposed model protects the sematic information of the training dataset in a distributed learning environment. The model was tested and analyzed by considering both the IID and Non-IID settings of the COVID-19 dataset. The experimental results have shown 3% increase in the overall performance compared to the FL model by ensuring the privacy of data. Similarly, Ho et al. (20) introduced a privacy-focused FL system for COVID-19 detection, aiming to create a decentralized learning framework among multiple hospitals that does not need the transfer of actual patient data. The proposed framework ensures the privacy of patient data by incorporating differential privacy techniques such as DP stochastic gradient descent (DP-SGD). The experimental results show that incorporating a spatial pyramid pooling layer into a 2D CNN, as well as specific design choices for handling Non-IID data, such as the number of total clients, the degree of client parallelism, and the computations per client, resulted in an increase in overall accuracy.

To achieve privacy with high utility in a distributed learning environment, the authors Li et al. (21) have proposed a secure Asynchronous Federated Learning (AFL) with DP algorithm for collaborative edge-cloud devices. The multi-stage adjustable private algorithm of the proposed model will dynamically adjust the noise and learning rates to improve the efficiency and convergence. The experimental findings show better results compared to the existing machine learning models with improved privacy. Lu et al. (22) has proposed a differentially private AFL approach for data sharing in vehicular networks. The authors have proposed local DP technique to nullify the attacks caused by the centralized curator during the weighted aggregation process. The experimental results have shown faster convergence with a few observations as the number of clients’ increases such as increased training period required to learn from the server model with reduced accuracy. Nguyen et al. (23) has proposed a novel asynchronous federated optimization framework with buffered asynchronous aggregation and Differential privacy scheme. The model was aimed to achieve improved privacy and scalability. The simulation results of the model outperformed the traditional methods.

Li et al. (24) have proposed an optimized asynchronous federated model for a depression detection system. The model was designed to enhance both the communication efficiency and the convergence rate while maintaining users’ privacy using the DP technique. The experimental results have shown 86.67% accuracy and minimal communication cost. Even though the FL provides a privacy guarantee for the user’s data, to strengthen the privacy safeguards the authors, Nampalle et al. (25) have proposed a novel FL with a DP technique for medical image classification. The proposed method consists of a novel noise calibration mechanism and adaptive privacy budget allocation strategy. Even though the simulation results have shown an improved efficiency in the classification of skin lesions and brain tumor images, the model requires further analysis and testing to improve the overall performance. Malik et al. (26) introduced DMFL_Net, a FL-based model for COVID-19 image classification. The study aims to improve COVID-19 classification, data privacy, and communication efficiency across medical institutions. The model incorporates DenseNet-169 into FL environment to enable collaborative training without sharing its contents to clients, thus guaranteeing privacy. The experiments were conducted on chest X-ray images to compare the performance of DMFL_Net with the conventional transfer learning approaches VGG-19 and VGG-16. The experimental results show that the proposed DMFL_Net model attains an accuracy of 98.45%, outperforming all other models and ensuring data privacy and optimal communication efficiency between participating hospitals. Dayan et al. (27) proposed a FL model named EXAM, that predicts the future oxygen requirements for COVID-19 patients based on chest X-rays, vital signs, and test results. The primary objective of the present study is to design a robust, generalizable model that can classify patients efficiently and effectively among different healthcare systems without the need for personal information sharing, thereby enhancing privacy and data security. The proposed model utilizes a 34-layer CNN (ResNet34) for extracting features from chest X-rays and a Deep & Cross network for integrating EMR features. The experiments were performed on data collected from 20 institutes around the world, and the results indicate that the proposed EXAM model enhanced accuracy and generalizability across trained models, with an AUC increase of 16 and 38% for generalizability.

Table 1 represents the summary of existing differential privacy-based Federated Learning models.

**Table 1:** Summary of existing DP-based FL models.

References	Methodology	Advantages/salient feature	Disadvantages/future enhancement
Chen et al. (13)	“DP Based adaptive worker selection algorithm for FL with LSTM training model.”	Resolves the issues of inadequate amount of dataset, ensure users data privacy using DP mechanism	Requires further threat analysis.
Wu et al. (14)	Adaptive gradient descendent mechanism with DP for collaborative learning	The model shows strong robustness and is less volatile.	The model suffers from convergence issues for a large set of data.
Ulhaq and Burmeister (15)	FL-based DP model for disease diagnosis.	Seven design principles are defined for effective implementation.	Only a theoretical model, hence it requires actual implementation for proper analysis
Wang et al. (16)	FL model with variational autoencoder (VAE) and DP preserve the patient’s data privacy	The model guarantees high accuracy and low adversarial inference attacks	Lack of strategies to improve the accuracy of a global model.
Liu et al. (17)	Hybrid Differential Privacy Model for FL.	The model removes the adverse effect of noise addition by using the adaptive clip method	Lack of strategies to stabilize correctness, privacy, and communication in FL
Zhang et al. (19)	GAN-based DP mechanism for FL (FedDPGAN). GAN Based DP mechanism for FL (FedDPGAN).	High-quality training samples generation.	High-quality training samples generation.
Ho et al. (20)	FL-based DPSGD for disease analysis, CNN model incorporating a spatial pyramid pooling strategy.	Improved robustness of the Model and improved accuracy of Non-IID data.	The model requires further analysis by considering a large dataset.
Nampalle et al. (25)	Adaptive privacy budget allocation mechanism for FL.	Improved privacy of medical data.	The proposed model failed to harmonize privacy and model performance
Malik et al. (26)	DMFL_Net for the classification of COVID-19	High classification accuracy and robustness in privacy preservation.	The FL model’s complexity limits its ability to scale to larger networks of organizations.
Dayan et al. (27)	FL for predicting clinical outcomes COVID-19 patients	The use of FL improved accuracy and privacy, making it appropriate for sensitive medical applications.	Due to the complexity of managing and synchronizing updates across the network, it does not scale smoothly as the number of participating sites increases.

**Figure 2:**
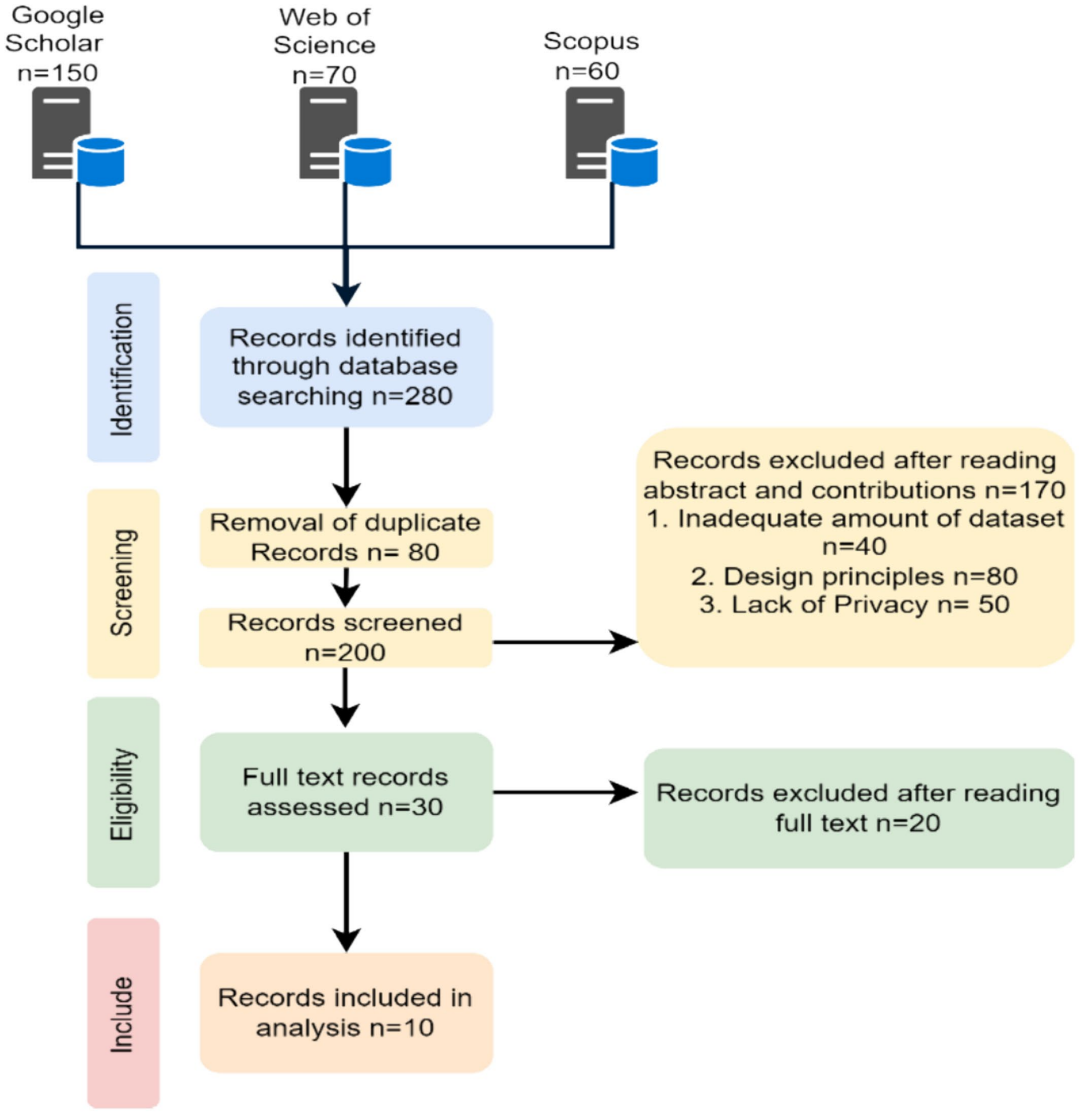
Prisma flow chart.

The literature review for Section 2 was carried out in accordance with the PRISMA guidelines shown in Figure 2.

## 3 Proposed model

In this section we present the preliminaries of Federated average algorithm and differential privacy mechanism. Following that, we present an overview of our proposed model, including the architecture and approaches used to classify Chest X-ray images to identify COVID-19 cases.

### 3.1 Differential privacy

Differential privacy (DP) enables the analysis of the features of an entire dataset or population without disclosing any personal information. A differentially private algorithm ensures that the inclusion or exclusion of a tuple from the dataset has no vital effect on the output. Dwork et al. defined DP as follows:

Definition 1: (ϵ,δ) —Differential Privacy—“A randomized algorithm *R:J → K* with input domain J and output range *K* is (ϵ,δ) -differentially private if for all pairs of neighboring datasets J, $J^{\prime }\in J$ , and every measurable L⊆K , we have $\mathrm{Pr}\left(R\left(J\right)\in L\right)\leq e^{\varepsilon }\cdot \mathrm{Pr}\left(R\left(J^{\prime }\right)\in L\right)+\delta$ where probabilities are with respect to the coin flips of *R* Equation.”

Where the privacy budget ϵ is used to determine the strengths of privacy protection and δ=0 result in ϵ -differential private mechanism. This type of DP is accomplished by introducing noise, which is identified through a sensitivity analysis of the dataset. Lower values of ε improve privacy but reduce effectiveness because of more noise, which lead to poor accuracy. Higher ε values improve data utility while compromising privacy. The chance of a further privacy violation after the ε guarantee is controlled by a measure called δ. When adjusting ε and δ, we must consider the desired prediction accuracy, acceptable privacy risk, and data sensitivity.

The following two probabilistic methods help to induce noise.

Laplace mechanism (10): The Laplace mechanism is a process of adding noise derived from the continuous Laplace distribution $\left(0,\frac{\Delta_{p}}{\epsilon }\right)$ where $\Delta_{p}$ is the sensitivity of function p, which measures the largest change in function p’s output generated by adding or removing a single individual’s data from the dataset. A higher sensitivity indicates that the function is more responsive to changes in the input dataset. During the process of noise addition to the dataset, L1 sensitivity and the epsilon value (i.e., the privacy budget) are considered for effective results. Hence, the Laplace mechanism can be defined as below:

Definition 2: “Given a function $p:J^{i}\to Y$ , where Y is the set of all possible outputs, and ϵ > 0.” The Laplace mechanism is represented in Eq. (1).


(1) $$R\left(J\right)=p\left(J\right)+Lap\left(0,\frac{\Delta_{p}}{\epsilon }\right)$$

Gaussian mechanism (10): The Gaussian Mechanism is a substitution to the Laplace Mechanism, which adds Gaussian Noise and supports tractability of the privacy budget under composition. Unlike Laplace Mechanism, Gaussian Technique uses L2 sensitivity rather than the L1 sensitivity, providing better control over the privacy budget by ensuring reasonable privacy guarantees and smoother noise distribution of L2 sensitivity will also preserve the utility. It can be defined as below.

Definition 3: "Given two neighboring datasets J and J’ in the dataset universe $J^{i}$ , a query function $p:J^{i}\to G$ , where G is the set of all possible outputs, and ϵ > 0″. The ϵ -Gaussian DP ( ϵ -GDP) mechanism is given in Eq. (2).


(2) $$R\left(J\right)=p\left(J\right)+N\left(0,\frac{\Delta_{p}^{2}}{\epsilon^{2}}\right)$$

Where, $N\left(0,\frac{\Delta_{p}^{2}}{\epsilon^{2}}\right)$ is considered as the normal distribution.

### 3.2 Federated averaging process

In a FL system that includes one server and *n* clients, where each client maintains local database *J_i_* where *i* = *{1, 2, 3,…,n}*. The server’s objective is to continuously learn from the data stored on *n* clients through multiple iterations, employing the local weights sent by the *n* clients to minimize loss. The optimization problem can be represented as shown in Eq. (3).


(3) $$Wt^{*}=\arg\underset{Wt}{\min}\overset{n}{\underset{i=1}{\sum }}p_{i}F_{i}\left(Wt,J_{i}\right)$$

Here, Wt^*^ denotes the server model parameter generated after aggregating the local models from *n* clients, *Wt_i_* is denoted as the model parameter from the *i*th client, and F_i_ is considered as the loss function of the *i*th client. Overfitting to specific client datasets in a heterogeneous data environment is a challenge in FL. Regularization and model averaging methods are used to address this issue. Applying regularization to the loss functions $F_{i}$ helps in minimize overfitting, and Federated Averaging engages averaging model updates from clients to reduce overfitting. $p_{i}$ is proportional to the amount of data $J_{i}$ contained by client *i*, affecting the client total model. The value of $p_{i}$ impacts the convergence rate of the model. Managing these weights is essential for guaranteeing that the model performs well among all client data transfers. The training mechanism of FL systems consists of several steps: Initially, the FL model sets the server’s weights. After that, it executes the following steps over multiple rounds:

**Step 1:** Forwarding the server weights: Server weights are forwarded to N clients in a network. Later, each client keeps a buffer to store the received weights in multiple iterations for future reference.
**Step 2:** Client Model Training: Using the latest model sent by the server, the clients will train their data on local machines. Soon after the training process, the updated models are returned to the server for further operations.
**Step 3:** Client Model Aggregation: The updated client model weights from *n* clients are transferred to the server. Later, the server will generate new weight by aggregating all client weight updates through mean computation, which is represented in Eq. (4).

(4) $$Wt^{\prime }=\frac{\sum_{i=0}^{n}Wt_{i}}{\sum_{i=0}^{n}i}$$

### 3.3 DP enabled federated averaging algorithm

In this section, we will discuss the architecture and steps involved in the proposed DPFL model and the pseudocode of the DPFL.

#### 3.3.1 Model architecture

The proposed DP-based FL model is aimed at providing user-level privacy by modifying the basic Federated Average algorithms in two different ways:

1 Clip the Model Updates: Model clipping is performed using adaptive methods instead of predefined clipping norms. The adaptive approach updates the clipping threshold based on a specific quantile, ensuring that values are accurately estimated within that range. Also, enables the model to maintain stability and convergence while effectively controlling the magnitude of updates, aimed to improve training performance and model accuracy.

Let A∈S be a random variable and β∈ *[0,1]* be a quantile to be satisfied. Then, for any T is given in Eqs. (5, 6) results in Eq. (7).

(5) $$l_{\beta }\left(\mathrm{T;A}\right)=\{\begin{array}{c} \left(1-\beta \right)\left(T-A\right)\;\mathrm{if}\;A\leq T\\ \beta \left(A-T\right)\;\mathrm{otherwise}\; \end{array}$$

So

(6) $$l_{\beta }^{\prime }\left(\mathrm{T;A}\right)=\{\begin{array}{c} \left(1-\beta \right)\;\mathrm{if}\;A\leq T\\ -\beta \;\mathrm{otherwise}\; \end{array}$$

Hence,

(7) $$\begin{array}{l} E\left[l_{\beta }^{\prime }\left(\mathrm{T;A}\right)\right]=\\ \;\;\;\;\;\left(1-\beta \right)\mathrm{Pr}\left[A\leq T\right]-\beta Pr\left[A>T\right]=\mathrm{Pr}\left[A\leq T\right]-\beta \end{array}$$

**Figure 3:**
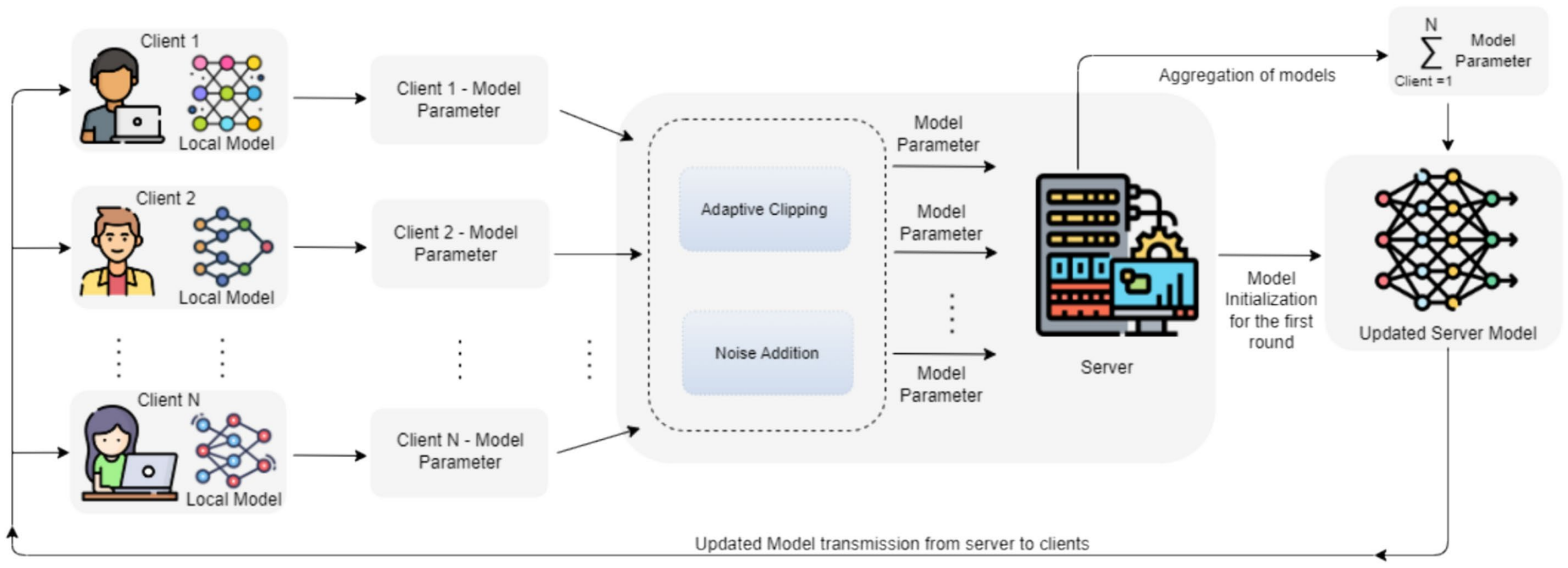
Stages of proposed DPFL model.

2 Addition of noise: In order to improve privacy without degrading the utility of data, the proposed model will be monitored using the standard deviation of the Gaussian noise and number of clients. Initially, we determine the noise tolerance of the model based on a varied amount of noise values by considering a small number of clients per round. Then we train the final model with increased noise on the sum and more clients per round. Reducing the number of clients at first eases the computational load and allows for effective noise level exploration. This methodology facilitates the assessment of the impact of varying noise levels on the usefulness of the information while offering valuable perspectives on the balance between privacy and usefulness. Figure 3 depicts the stages of the proposed DPFL model.

#### 3.3.2 DPFL algorithm

Considering *n* as the number of users in a round and β∈ *[0,1]* as the target quantile for the norm distribution where clipping is to be applied, for every iteration m∈[M] , let $V^{m}$ represent the clipping threshold, and $\eta_{V}$ the learning rate. Let $Y^{m}$ be the set of users sampled in round *m*. Each user $k\in Y^{m}$ will send the binary indicator $a_{k}^{m}$ along with the usual model update $\Delta_{k}^{m}$ , where $a_{k}^{m}=I_{\left(\Delta_{k\;2}^{m}\leq V^{m}\right)}$ . Defining ${\bar{a}}^{m}=\frac{1}{n}\underset{k\in Y^{m}}{\sum }a_{k}^{m}$ , we apply the update $V\leftarrow V\cdot \exp\left(-\eta_{V}\left(\bar{a}-\gamma \right)\right)$ However, to prevent the leakage of private information through model updates, we add Gaussian noise to the sum ${\tilde{a}}^{m}=\frac{1}{n}\left(\underset{k\in Y^{m}}{\sum }a_{k}^{m}+N\left(O,\sigma_{a}^{2}\right)\right)$ .

The target quantile (β) for the normal distribution affects the clipping threshold (V^m^) by selecting the value at which the distribution’s tails are trimmed. Higher β values result in higher clipping thresholds, allowing for further removal of the distribution. The learning rate $\eta_{V}$ in the update rule for V controls how quickly the clipping threshold adjusts to observed gradients. Higher $\eta_{V}$ results in quicker V modifications, potentially speeding up convergence by allowing the model to react to changes in data distribution. Excessive $\eta_{V}$ values disrupt training, leading to divergence. A lower $\eta_{V}$ promotes stability but delay convergence rates. The regularization parameter γ maintains the clipping threshold within the intended bounds by modifying it in response to the discrepancy between the target value γ and the average clipping rate $\bar{a}$ . Thus, the federated learning process’s privacy-utility trade-off is adjusted by varying γ. Algorithm 1 depicts DPFL Algorithm.

##### ALGORITHM 1 : DPFL Algorithm

**Table:** 

*Function Train* $\;\left(n,\beta ,\eta_{v},\eta_{z},\eta_{V},x,\sigma_{a},\beta \right)$		
*Initialize model* $\theta^{0}$ *, clipping bound* $V^{0}$		
$x_{\Delta }\leftarrow {\left(x^{-2}-{\left(2\sigma_{b}\right)}^{-2}\right)}^{-1/2}$		
***For** (each round m = 0,1,2, ………) **do***		
	$Y^{m}\leftarrow \left(sample\;m\;users\;uniformly\right)\;$	
	***For** each user* $k\in Y^{m}$ *in parallel **do***	
		$\left(\Delta_{k}^{m},a_{k}^{m}\right)\leftarrow FedAvg\;\left(k,\theta^{m},\eta_{v},V^{m}\right)$
	***End For***	
	$\sigma_{\Delta }\leftarrow x_{\Delta }V^{m}$	
	${\tilde{\Delta }}^{m}=\frac{1}{n}\left(\underset{k\in Y^{m}}{\sum }\;\Delta_{k}^{m}+N\left(0,I\sigma_{\Delta }^{2}\right)\right)$	
	${\bar{\Delta }}^{m}=\beta {\bar{\Delta }}^{m-1}+{\tilde{\Delta }}^{m}$	
	$\theta^{m+1}\leftarrow \theta^{m}+\eta_{z}{\bar{\Delta }}^{m}$	
	${\tilde{a}}^{m}=\frac{1}{n}\left(\underset{k\in Y^{m}}{\sum }\;a_{k}^{m}+N\left(O,\sigma_{a}^{2}\right)\right)$	
	$V^{m+1}\leftarrow V^{m}\cdot \exp\left(-\eta_{V}\left({\tilde{a}}^{m}-\beta \right)\right)$	
***End For***		
$function\;FedAvg\;\left(i,\theta^{0},\eta ,V\right)$		
$\theta \leftarrow \theta^{0}$		
$B\leftarrow \left(user\;k^{\prime }s\;local\;data\;is\;divided\;into\;batches\right)$		
Forbatchb∈B ***do***		
	θ←θ−η∇l(θ;b)	
	$\Delta \leftarrow \theta -\theta^{0}$	
	$a\leftarrow I_{∥\Delta ∥\leq V}$	
	$\Delta^{\prime }\leftarrow \Delta \cdot \min\left(1,\frac{V}{∥\Delta ∥}\right)$	
***End For***		
$return\;\left(\Delta^{\prime },a\right)$		

### 3.4 Early stopping mechanism

The early stopping technique is a widely utilized method for regularization in DNN. It is an effective and simple technique that typically outperforms most of the general regularization approaches. During training, the model continually stores and updates the best parameters attained so far. If there’s no further improvement in validation error after a set number of iterations, the training halts, retaining the last best parameters. When dealing with models that are prone to overfitting, it is common to recognize a gradual decrease in training error followed by an increase in validation error. Early stopping represents a balance between training duration and generalization error, minimizing communication overhead while still achieving optimal parameters. By reducing the need for communication and subsequently diminishing noise, early stopping enhances the utility of the data. The early stopping algorithm can be represented in Algorithm 2 as follows:

#### ALGORITHM 2 : General Early Stopping Mechanism

**Table:** 

***Input: s➔** represents the number steps during the evaluation period.*		
***e➔** represents the number of epochs, meaning it terminates after observing the worse performance.*		
$\theta_{0}$ *➔ represents the initial parameter.*		
$\theta \leftarrow \theta_{0}$		
p←0		
$q\leftarrow 0,r\leftarrow \infty ,\theta^{*}\leftarrow \theta ,p^{*}\leftarrow p$		
***While*** ( q<e ) ***do***		
	*Execute the training algorithm for s steps and update* θ	
	p←p+n	
	$\;\;r^{\prime }\leftarrow$ *validation_set_error* (θ)	
	***If*** $r^{\prime }<r$ *then*	
		$q\leftarrow 0,\;\theta^{*}\leftarrow \theta ,\;\;p^{*}\leftarrow p,\;\;r\leftarrow r^{\prime }$
	***Else***	
	q←q+1	
	***End If***	
***End while***		
**Output:** The optimal parameter $\;\theta^{*}$ , the optimal number of training steps $p^{*}$		

## 4 Experimental results

This section discusses the experimental activities used to analyze and evaluate the effectiveness of the proposed algorithm. We discuss the dataset, experimental setup, model and training data, and performance analysis using various metrics.

### 4.1 Dataset description

**Figure 4:**
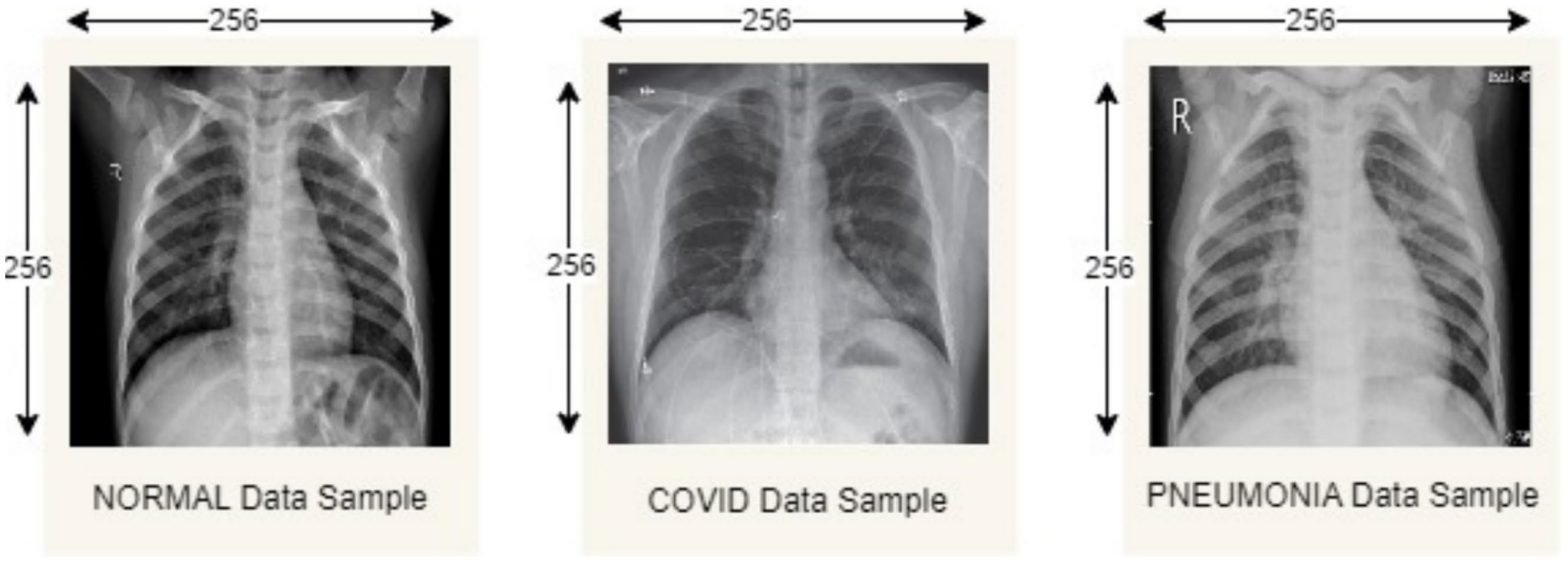
Normal, COVID19, pneumonia chest X-ray image samples.

The proposed model is evaluated considering the Covid19, Pneumonia, Normal Chest X-Ray Image dataset from Mendeley Data (28). This dataset includes 5,228 chest X-ray images categorized into three categories: 1,626 COVID-19, 1,802 normal (asymptomatic), and 1,800 pneumonia (non-COVID-19). All images are resized to 256 * 256 pixels to reduce computational load, which is important in a FL environment where computations are distributed across devices of different capabilities. During the process we classify the image dataset into train and test sample datasets having 4,182 training samples and 1,046 testing samples, respectively. Table 2 describes the data distribution among each of the categories, and Figure 4 depicts sample images from each category.

**Table 2:** Distribution of the COVID-19 dataset into training and testing sets.

Data-split details	Normal	Covid-19	Pneumonia
Train data samples	1,442	1,300	1,440
Test data samples	360	326	360

### 4.2 Implementation and model

**Figure 5:**
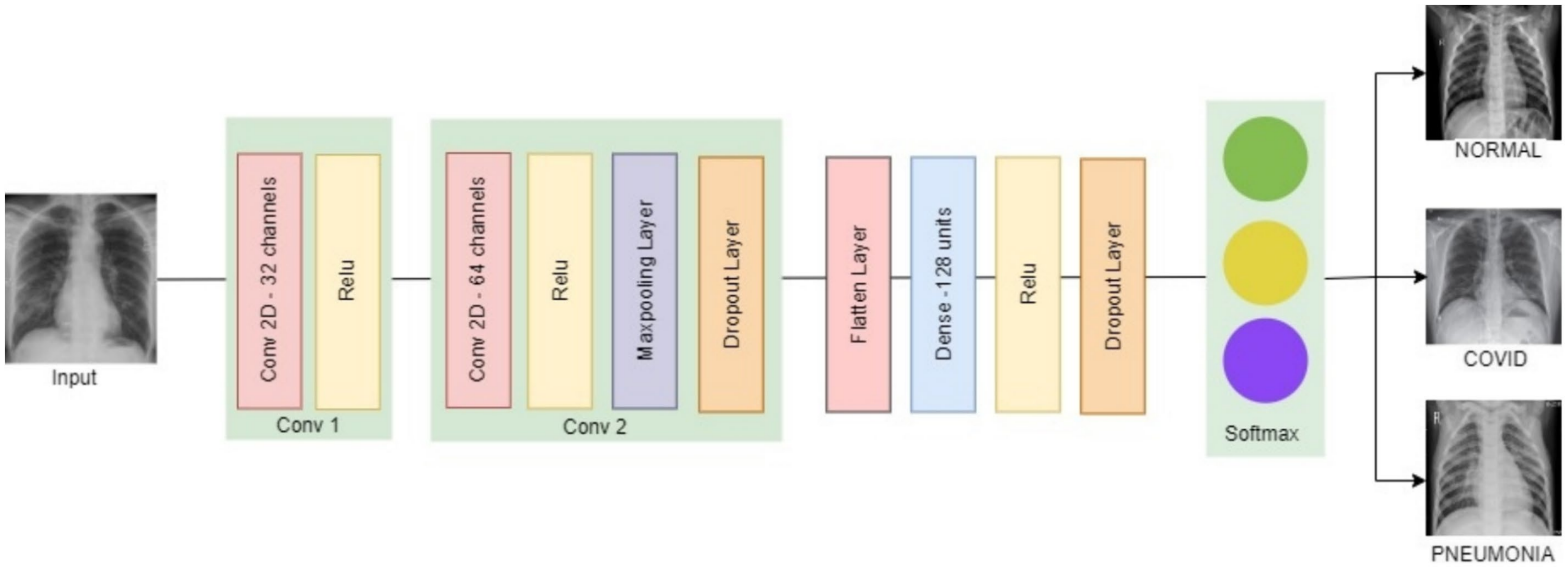
CNN model architecture.

The proposed model is developed using the Python programming language and evaluated within a Tensorflow framework in a Colab environment. TensorFlow Federated and TensorFlow Privacy packages allow developers to simulate and test the functioning of distributed learning with privacy. TensorFlow Federated provides a wide range of FL-specific features. This allows for the modeling of FL processes on decentralized data, which is crucial for our research as data privacy and local computation are essential. The TensorFlow Privacy framework includes pre-built mechanisms, such as optimizers, to make it easier to integrate differential privacy into machine learning processes. The primary objective is to categorize the disease into three groups: normal, COVID-19, and pneumonia, through the use of CNN model. Our CNN model, depicted in Figure 5, contains two 3 × 3 convolutional layers with 32 and 64 channels, followed by a 2 × 2 max pooling layer. The two convolutional layers were used to achieve a balance between model complexity and computational efficiency, which is important in a FL environment where edge devices have limited computational resources. It includes a fully connected layer with 128 units and utilizes ReLU activation, a softmax output layer for classification. To prevent overfitting during the training process, two dropout layers with probabilities of 0.25 and 0.5 are positioned just before and after the fully connected layer.

### 4.3 Distributed and central architecture

**Figure 6:**
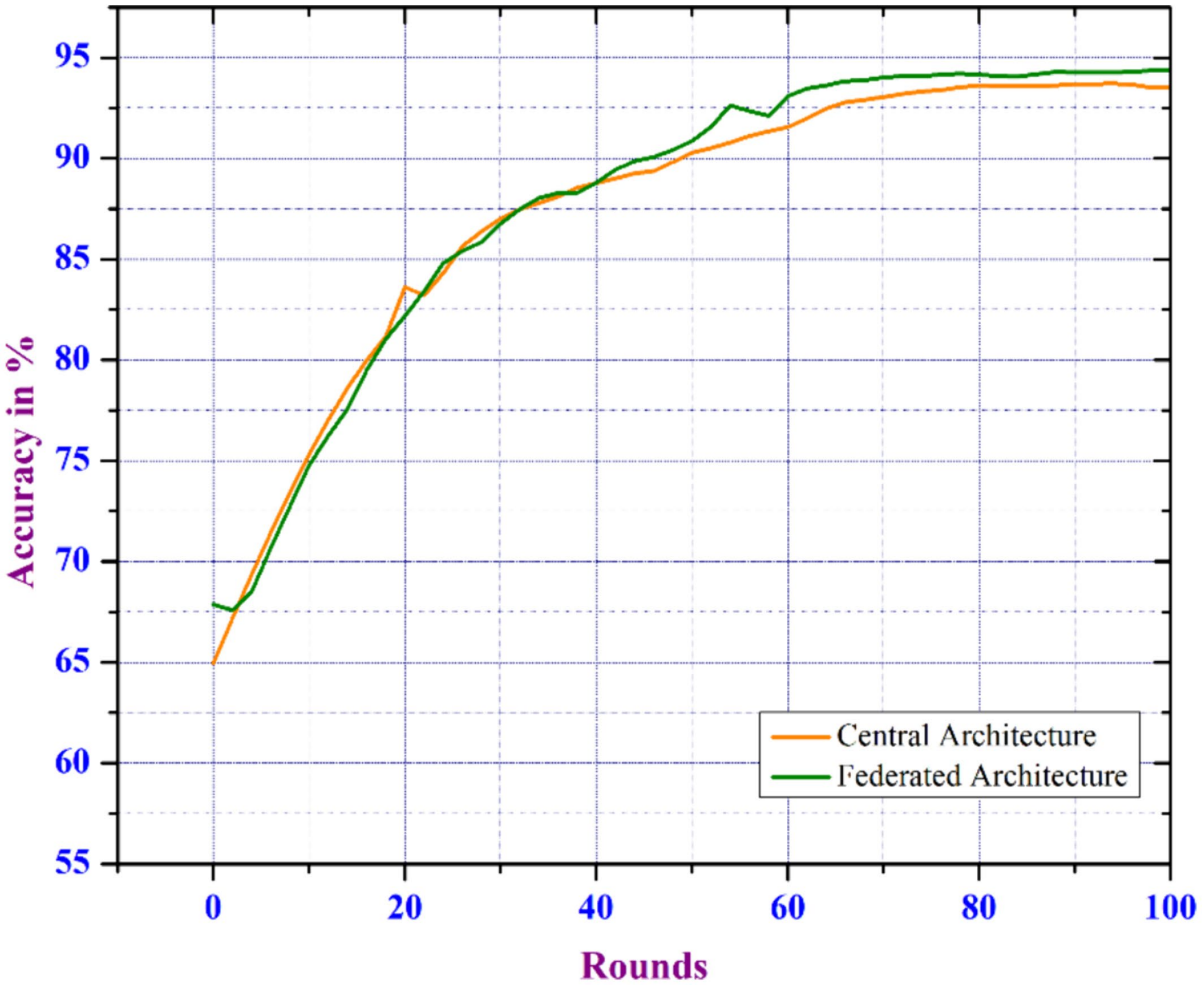
Comparison of model accuracy over communication rounds for central and federated learning architectures.

The CNN model is trained in both distributed and traditional central learning environments considering the parameters as number_of_clients = 100, client_ratio = 0.3, local_epochs = 2, and batch_size = 16. With the increase in number of rounds, the accuracy in identifying COVID-19 diseases enhances more in FL-based environments. Therefore, the FL model shows superior learning capabilities compared to conventional learning systems. The FL-based model performs better after 50 rounds of execution. Therefore, the overall accuracy of the FL-based approach achieves 94.3%, while central learning is 93.5%. Figure 6 depicts an analysis of communication rounds between FL and central learning models, indicating that training on diverse datasets from various clients results in better model generalization. In FL, the client trains a model using local data and only shares model updates. This minimizes the risk of overfitting for COVID-19 patient data. Each round of FL training provides new updates from multiple client datasets, improving the model’s ability to predict and achieve higher accuracy. This finding highlights distributed learning’s advantage over traditional central learning methodologies in terms of improving model performance.

The proposed distributed learning techniques are further evaluated by comparing various existing CNN models such as Resnet18, Resnet50, and VGG18, with our model. The analysis uses number_of_clients = 100, client_ratio = 0.3, local_epochs = 2, and batch_size = 16. Our CNN has an optimal number of layers, and activation functions that handle the data’s features more efficiently.

**Figure 7:**
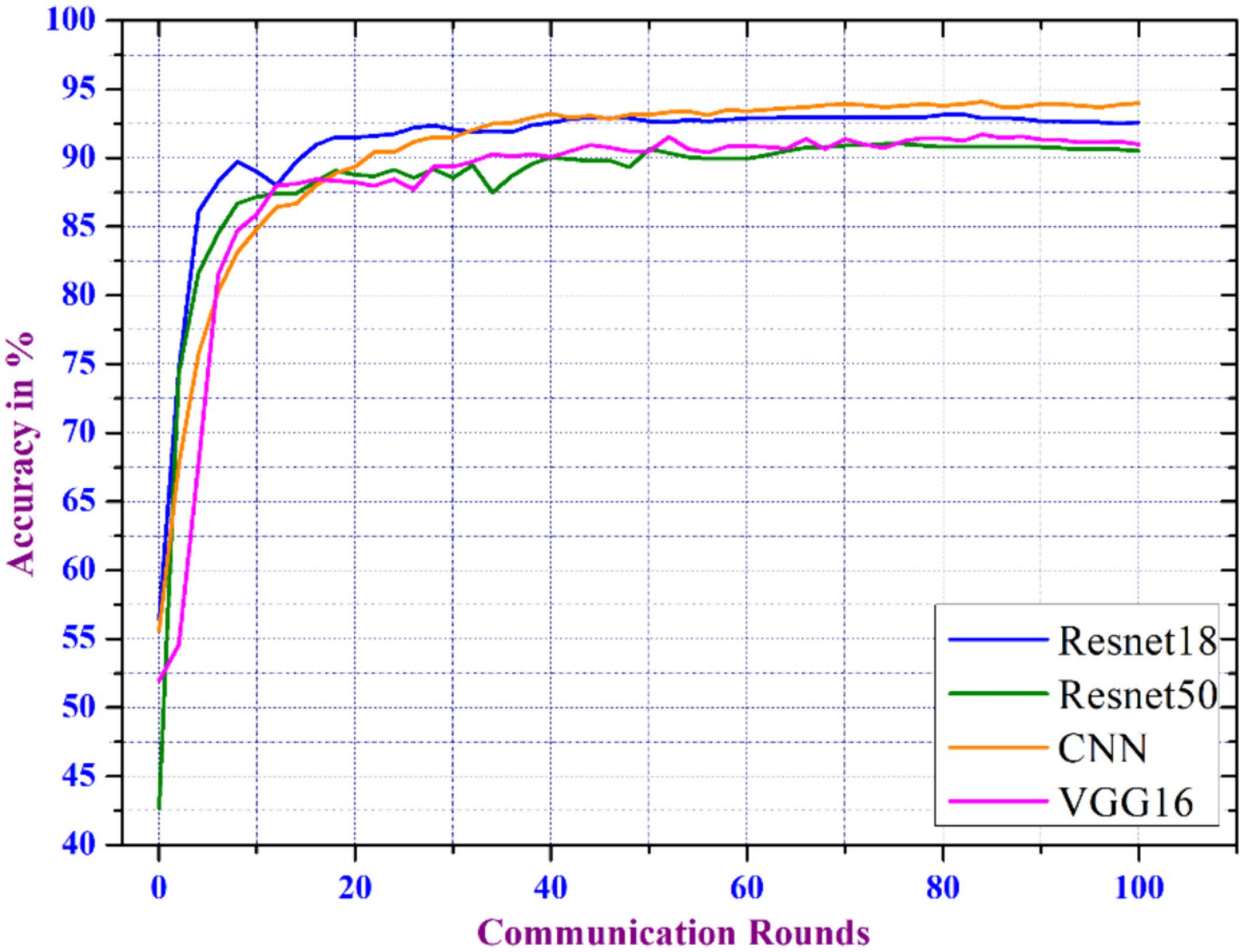
Comparative accuracy performance of CNN model against standard CNN architectures.

The model is designed to generalize better when trained on decentralized datasets and is highly parameter-efficient, resulting in higher accuracy with less parameters. This efficiency is important in FL, where models are updated throughout networks using minimal computational resources. Figure 7 depicts the accuracy analysis of the models where the CNN model outperforms the aforementioned models in terms of accuracy for different communication round. The primary goal of FL is to manage communication rounds with the computational and communication overheads. Frequent updates result in faster convergence and higher accuracy. We noticed that as the number of rounds increased, the model’s accuracy enhanced, implying that more frequent updates benefit model performance.

**Figure 8:**
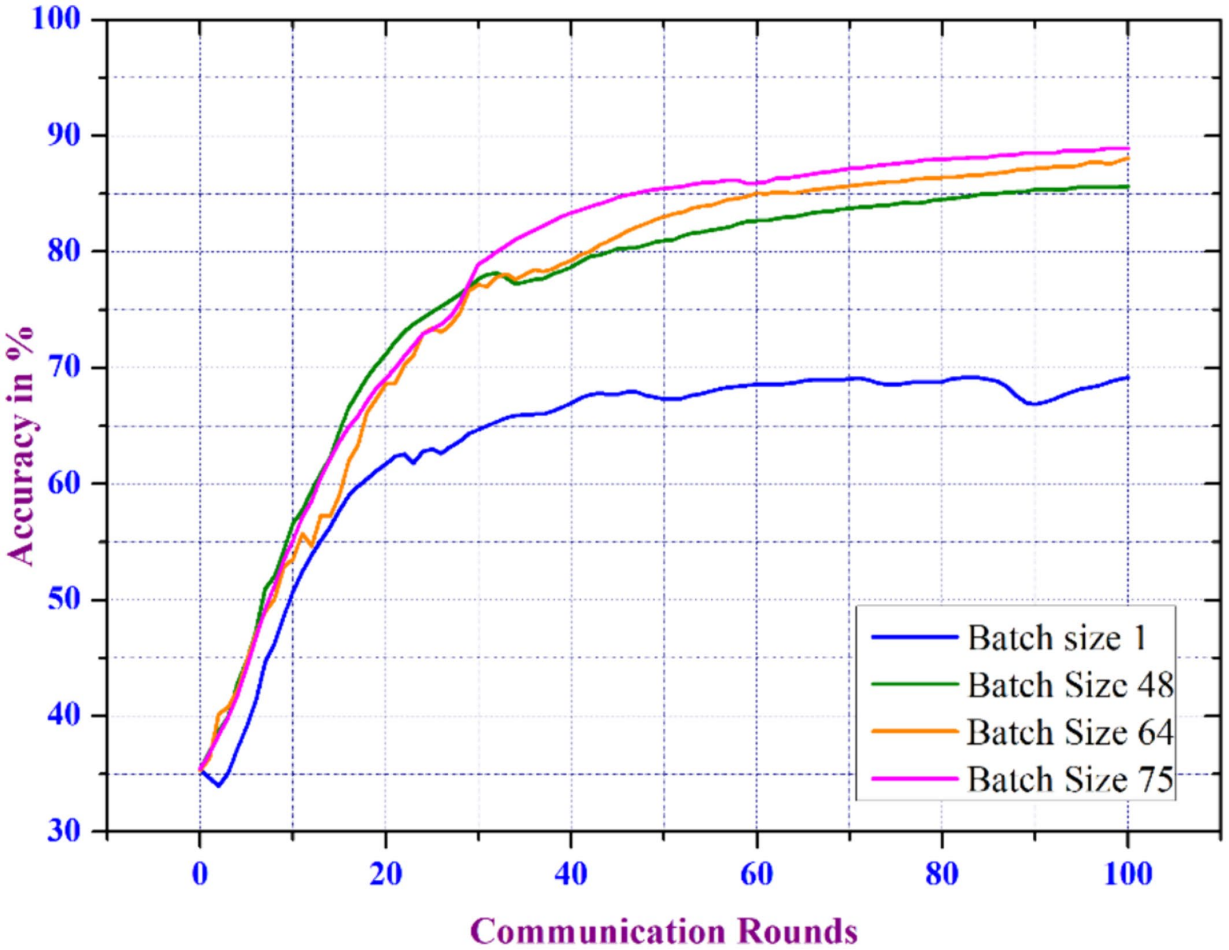
Accuracy analysis of FL model with respect to varied batch size.

The proposed distributed FL model undergoes additional analysis by varying the batch size, which shows that the FL model’s accuracy increases exponentially as the batch size increases across various rounds, as shown in Figure 8. Increasing the batch size leads to a larger volume of data processed during every round of training. Larger batch sizes help to smooth out noisy gradients and stabilize the training process, resulting in better convergence and accuracy. Therefore, this aids in enhancing the accuracy of the model’s learning process.

### 4.4 FL with differential privacy mechanism

**Figure 9:**
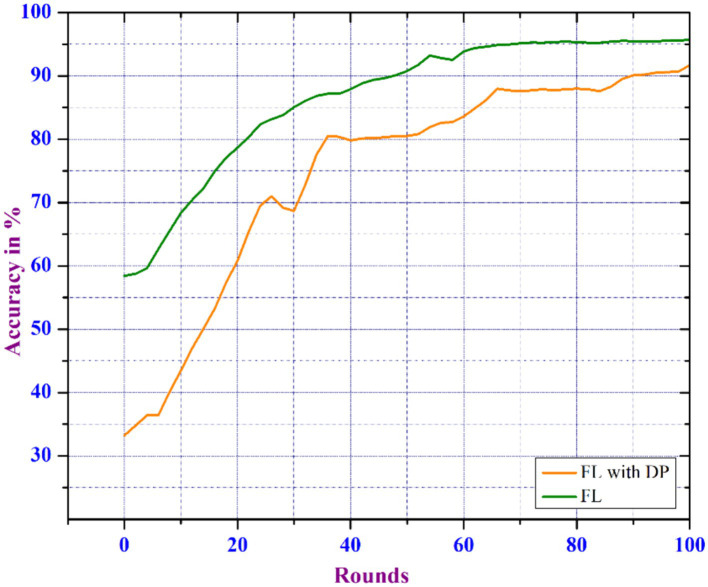
Comparison of FL vs. DP enabled FL.

FL guarantees privacy by eliminating the need to share data between participants or servers. To improve the privacy mechanisms of FL-based learning, we proposed the Differential Privacy Federated Learning model. The experiment is carried out in a distributed learning environment with a 0.2 noise_multiplier, 50 clients_per round, a learning_rate of 0.01, two epochs, and a client_ratio of 0.01. However, the introduction of noise reduces the accuracy of the DP-based FL when compared to the traditional FL. Figure 9 shows a 3% drop in accuracy for the DPFL-based model compared to FL. The noise disrupts the learning process, lowering the model’s capability to accurately capture the underlying patterns in the data. As a result, the introduced noise necessitates a compromise between privacy and model accuracy.

### 4.5 Model noise sensitivity analysis

Model Noise Sensitivity Analysis in FL is important for deploying FL models in environments where data noise is unavoidable, as it helps to understand how noise in the data affects the performance and reliability of learning models trained on various decentralized devices or servers. In the healthcare domain, the main focus is the accuracy of diagnosis models, as inaccurate predictions can have an immediate effect on the health of patients (29). However, because medical records are so sensitive, patient data privacy is a major concern (30, 31). To meet these requirements, healthcare professionals can select a lower noise multiplier if the model’s predictive accuracy is vital for critical diagnostic tasks. Yet, for less sensitive tasks, a higher noise multiplier may be sufficient to ensure more privacy. Our findings suggest a strategic approach in which noise levels are adjusted depending on the sensitivity of the data and the importance of the task. This enables health care professionals to keep patient trust by protecting their data while guaranteeing that the diagnostic models are as accurate as needed. Data scientists working in a variety of sectors particularly healthcare, are frequently challenged with creating models that balance usability and privacy standards. They could apply our findings to create adaptive privacy mechanisms that dynamically adjust the noise multiplier according to real-time assessments of data sensitivity and model performance. Understanding and minimizing the impact of noise can improve the reliability, accuracy, and effectiveness of FL models. To improve utility and maintaining privacy, our proposed model includes an adaptive clipping mechanism based on an increased noise addition mechanism. The adaptive clipping mechanism automatically adjusts the sensitivity between aggregated data as well model updates, resulting in an optimal balance of data privacy and model utility. This mechanism helps in controlling the impact of noise introduced to ensure privacy, improving the model’s learning efficiency, and protecting each data point. Initially, we train the model by considering 50 clients per round by considering noise multipliers in the range [0, 0.25, 0.5, 0.75, and 1.0].

**Figure 10:**
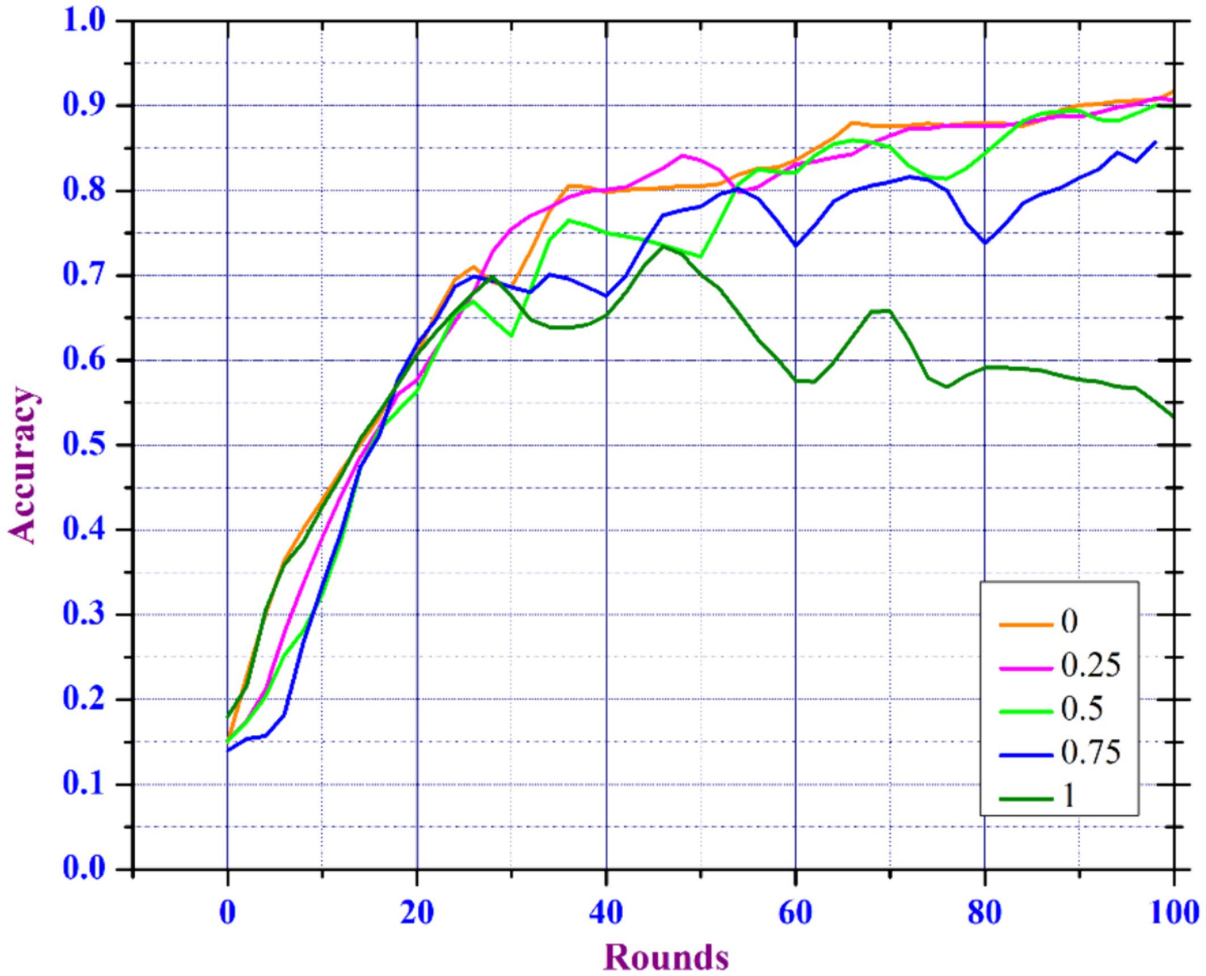
Accuracy analysis of DP enabled FL based on varied noise multiplier.

**Figure 11:**
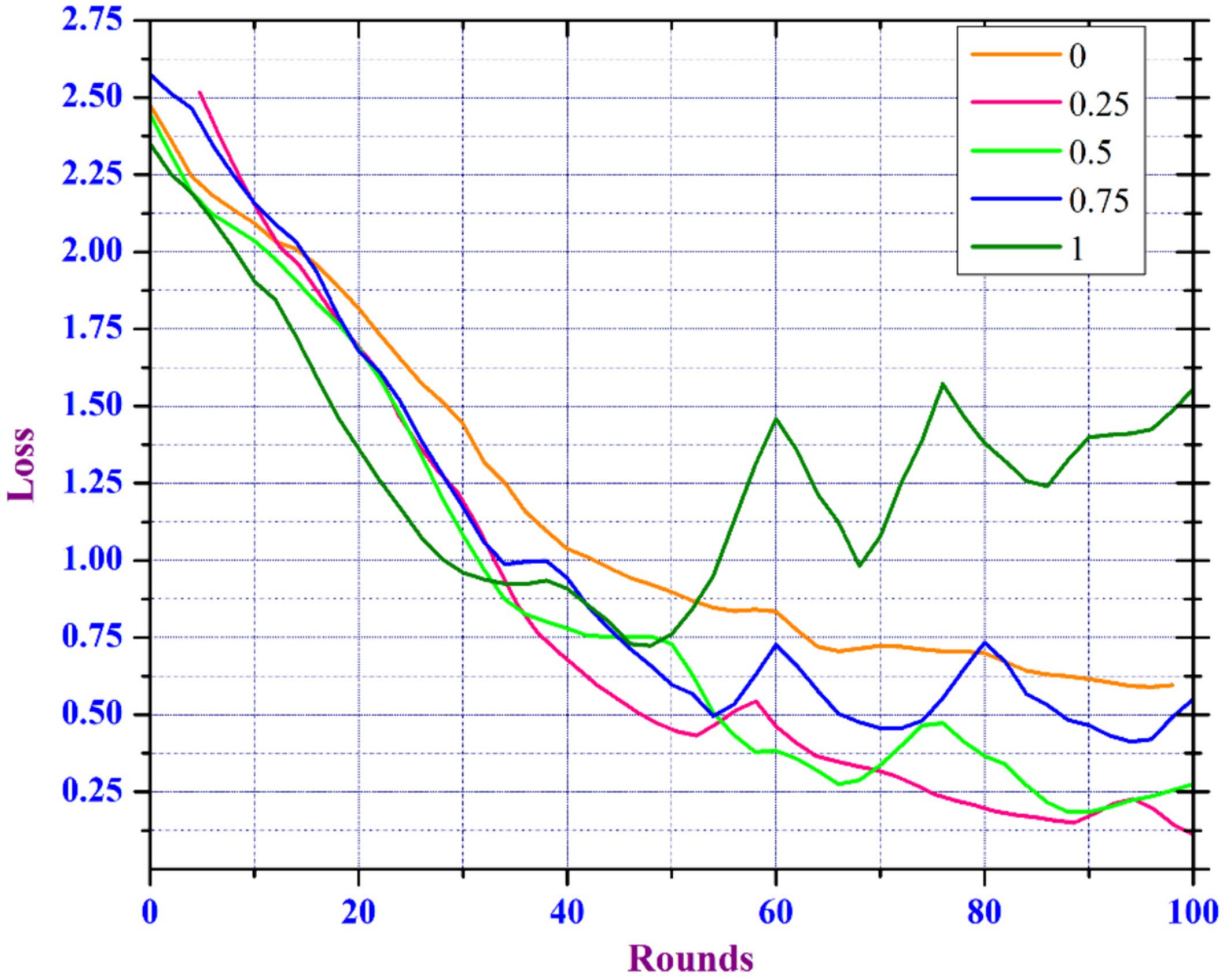
Loss analysis of DP enabled FL based on varied noise multiplier.

**Figure 12:**
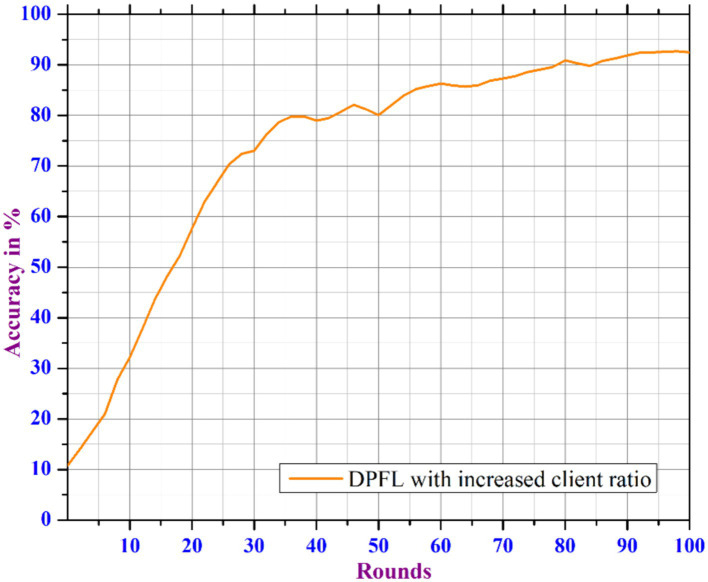
Accuracy analysis of DP enabled FL based on increased client ratio.

Figures 10, 11 show that the model can tolerate noise multipliers up to 0.5, implying that noise multipliers of 0, 0.25, and 0.5 do not decrease the utility of the data. However, a noise multiplier of 0.75 reduces accuracy, while 1.0 causes the model to completely diverge. The adaptive clipping mechanism allows the model to withstand noise up to a certain level (0.5 in this case) while maintaining utility. This demonstrates the effectiveness of the proposed method, which balances privacy and accuracy. Additional simulations are carried out to determine the implications of changing the client count in each round while keeping a constant noise multiplier of 0.25 and client ratio of 0.01 throughout the process. As the client count increased from 10 to 40, the model’s accuracy improved and the loss percentage decreased. However, based on the results of our previous experiments and with the goal of reducing data privacy risks while preserving data utility, we ran another simulation with a privacy budget of 1e-05 and a total of 120 clients per round. In spite of the increased noise multiplier, the outcomes show enhanced precision in comparison to earlier tests, suggesting that the privacy-preserving mechanisms successfully discover a balance between privacy and utility. Figure 12 depicts the improved accuracy of the proposed model. Therefore, increasing the number of clients per round results in a more diverse and representative dataset, resulting in better generalization and model efficiency.

### 4.6 Model performance for early stopping mechanism

**Figure 13:**
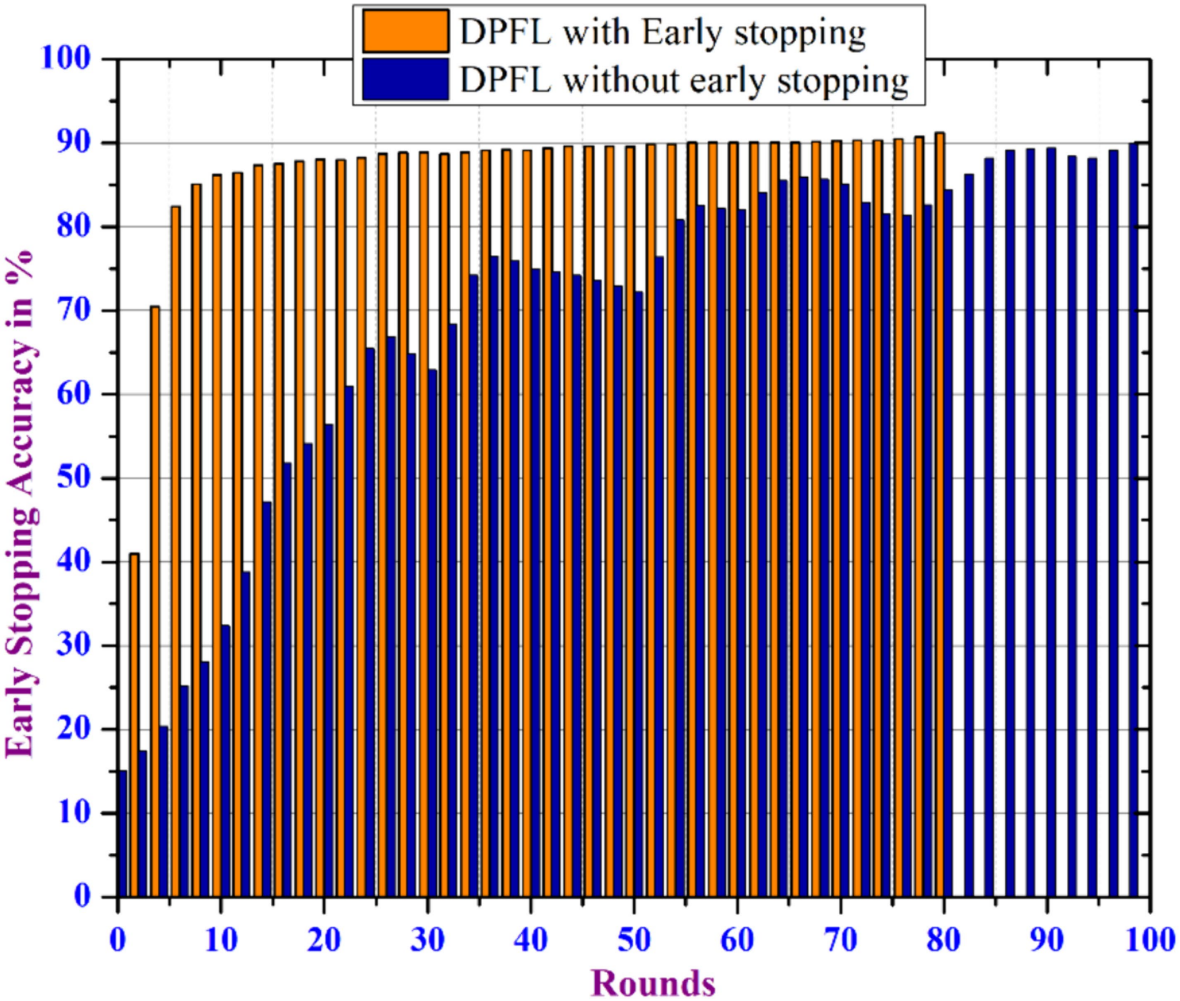
Accuracy analysis of early stopping mechanism.

Another experiment was carried out with a configuration of 50 clients_per round, a learning_rate of 0.01, and 100 epochs to investigate the impact of incorporating an early stopping mechanism into the proposed DPFL model, as shown in Figure 13. During the experiment, the proposed DPFL model’s accuracy improved as the number of training epochs increased by dynamically adjusting the noise range within a specific privacy level. By evaluating the model’s performance on a validation dataset during training, the early stopping mechanism terminate the training process when the model begins to overfit, thus improves the model’s generalizability. As a result, the integration of the early stopping mechanism with DPFL model achieved an accuracy of 91.2% after 80 epochs, hence it ensures the consistent privacy level throughout the training process, without sacrificing accuracy and also minimizes overall communication costs.

Early termination of training may have a disproportionate impact on specific clients, resulting in biased model updates and imbalances. This issue can be addressed by using the early stopping criterion based on client attributes or performance measures, ensuring that all clients contribute significantly to the training process and are treated equally.

## 5 Conclusion

In this work, we propose an enhanced Privacy-Preserving FL system with Differential Privacy techniques to predict COVID-19 using Chest X-Ray images. Initially, we trained Chest X-Ray image data using a CNN model, evaluating Federated and non-Federated training methods. The results show that FL-based training enhances performance by 0.8% over non-FL or traditional centralized learning. Secondly, we introduce an enhanced FL-based system that includes additional differential privacy and an adaptive noise inclusion mechanism. This system’s adaptive clipping effectively identifies the model’s noise tolerance level while preserving data utility across different noise scales. However, the proposed DPFL model’s initial results show a 3% reduction in accuracy when predicting COVID-19 due to the masking process. The integration of an efficient privacy-utility trade-off and an early stopping mechanism to DPFL has resulted in a 1% increase in accuracy and a decrease in communication rounds. As a result, the proposed early stopping-based DPFL model outperforms existing DP-based FL models in terms of COVID-19 predictions. The model can be further enhanced by considering the popular pre-trained models for a large dataset and also considering other aspects such as improving the scalability and robustness of the FL. Additionally the incorporation of various to techniques for model personalization, model generalization, and fair client contribution evaluation will further strengthen the model.

## Data availability statement

The original contributions presented in the study are included in the article/supplementary material, further inquiries can be directed to the corresponding author.

## Author contributions

RA: Conceptualization, Data curation, Investigation, Methodology, Software, Supervision, Writing – original draft, Writing – review & editing. PM: Conceptualization, Data curation, Formal analysis, Investigation, Methodology, Project administration, Software, Supervision, Validation, Writing – original draft, Writing – review & editing. TG: Conceptualization, Data curation, Investigation, Methodology, Software, Supervision, Writing – original draft, Writing – review & editing. NA: Conceptualization, Data curation, Funding acquisition, Methodology, Resources, Software, Visualization, Writing – original draft, Writing – review & editing. FH: Conceptualization, Formal analysis, Project administration, Resources, Software, Supervision, Validation, Visualization, Writing – original draft, Writing – review & editing.
