# Corrigendum: Efficient differential privacy enabled federated learning model for detecting COVID-19 disease using chest X-ray images

**DOI:** 10.3389/fmed.2024.1504309

**Published:** 2024-10-24

**Authors:** Rawia Ahmed, Praveen Kumar Reddy Maddikunta, Thippa Reddy Gadekallu, Naif Khalaf Alshammari, Fatma Ali Hendaoui

**Affiliations:** ^1^Computer Science Department, Applied College, University of Ha'il, Ha'il, Saudi Arabia; ^2^School of Computer Science Engineering and Information Systems, Vellore Institute of Technology, Vellore, Tamil Nadu, India; ^3^The College of Mathematics and Computer Science, Zhejiang A&F University, Hangzhou, China; ^4^Division of Research and Development, Lovely Professional University, Phagwara, India; ^5^Center of Research Impact and Outcome, Chitkara University, Rajpura, India; ^6^Mechanical Engineering Department, Engineering College, University of Ha'il, Ha'il, Saudi Arabia; ^7^Computer Science Department, Applied College, University of Ha'il, Ha'il, Saudi Arabia

**Keywords:** COVID-19 detection, decentralized training, adaptive differential privacy, federated learning, convolutional neural network, healthcare data privacy

In the published article, there was an error in Article Type “[SYSTEMATIC REVIEW article]”, it should be “[ORIGINAL RESEARCH article]”.

The authors apologize for this error and state that this does not change the scientific conclusions of the article in any way. The original article has been updated.

